# Medical Degrees for Women

**Published:** 1877-03

**Authors:** 


*Medical Degrees for Women.*—On March 2nd, 1877, the Senate of the University of London, England, decided by a vote of fourteen to eight, to admit women to medical degrees in that institution

From every section of the civilized world we almost daily learn of such priviliges being granted to the sex.

